# UV and Visible Spectrum LED Lighting as Abiotic Elicitors of Bioactive Compounds in Sprouts, Microgreens, and Baby Leaves—A Comprehensive Review including Their Mode of Action

**DOI:** 10.3390/foods11030265

**Published:** 2022-01-19

**Authors:** Francisco Artés-Hernández, Noelia Castillejo, Lorena Martínez-Zamora

**Affiliations:** Postharvest and Refrigeration Group, Department of Agronomical Engineering, Institute of Plant Biotechnology, Universidad Politécnica de Cartagena, 30203 Cartagena, Spain; noelia.castillejo@upct.es (N.C.); lorena.martinez@upct.es (L.M.-Z.)

**Keywords:** seed germination, ultraviolet, illumination, light-emitting diodes, abiotic stress, nutraceuticals, phytochemicals, health-promoting compounds

## Abstract

**Background:** According to social demands, the agri-food industry must elaborate convenient safe and healthy foods rich in phytochemicals while minimising processing inputs like energy consumption. Young plants in their first stages of development represent great potential. **Objective:** This review summarises the latest scientific findings concerning the use of UV and visible spectrum LED lighting as green, sustainable, and low-cost technologies to improve the quality of sprouts, microgreens, and baby leaves to enhance their health-promoting compounds, focusing on their mode of action while reducing costs and energy. **Results:** These technologies applied during growing and/or after harvesting were able to improve physiological and morphological development of sprouted seeds while increasing their bioactive compound content without compromising safety and other quality attributes. The novelty is to summarise the main findings published in a comprehensive review, including the mode of action, and remarking on the possibility of its postharvest application where the literature is still scarce. **Conclusions:** Illumination with UV and/or different regions of the visible spectrum during growing and shelf life are good abiotic elicitors of the production of phytochemicals in young plants, mainly through the activation of specific photoreceptors and ROS production. However, we still need to understand the mechanistic responses and their dependence on the illumination conditions.

## 1. Introduction

Horticultural products are the most important and most-studied foods as a source of nutraceutical compounds. In this sense, due to the richness of the Mediterranean diet in fruits and vegetables, this dietary pattern has been considered one of the healthiest and was declared an Intangible Cultural Heritage of Humanity by UNESCO in 2013 [1]. Furthermore, young plants specifically in their first stages of development as sprouts, microgreens, and baby leaves have been demonstrated having more than 20-fold bioactive compound content compared to adult plants [2,3]. This makes them an important source of phytochemicals with a great benefit to be included in our daily balanced diet.

In this context, over the past several decades, and according to consumer requests, conventional crops and agri-food industries have widely increased and developed their production chains to satisfy the food demands related to the global growth of the population [4]. Regarding the social concern, the promotion of a healthy diet and regular physical activity have increased the necessity of nutraceutical foods and ingredients, which can provide positive effects to the human body, specifically since the recent pandemic due to the SARS-CoV-2 virus [5].

Lighting is one of the main energy sources during plant development and plays an essential role in the biosynthesis of health-promoting compounds. To adapt food industries to the new era of contemporary climate change, avoiding economic and energy waste is an essential challenge to focus on in research and development. Therefore, reducing the energy consumption during lighting is an essential point to take into consideration.

To make lighting more efficient, light-emitting diodes (LEDs) have been developed, which emit light at different wavelengths when the energy flows throughout the semiconductor and releases it transformed into photons. Technological advances in this technology have resulted in relevant improvements in agriculture, where intensity and spectral properties can be customised to adapt them to optimise the crop yield [6]. Illumination with regions of the light spectrum (from ultraviolet (UV) to infrared) can influence the biosynthesis of phytochemicals in a different way, as well as improve the plant development at different stages of growth, elongation, flowering, or fruit-setting.

Therefore, physical elicitation produced by the light perceived can be controlled to trigger the activation of the plant secondary metabolism as a defence mechanism against light incidence through the use of LEDs, also stimulated by photoreceptors and genes that are activated against abiotic stresses [7].

As a matter of fact, many authors have confirmed the relation between the stress generated by lighting and the increase in the biosynthesis and accumulation of nutraceutical compounds. For instance, low doses of UV-B and UV-C have been demonstrated to be good elicitors of the biosynthesis of carotenoids and flavonoids in bell peppers [8], as well as of glucosinolates and isothiocyanates in broccoli [2,9], radish sprouts [10], and kale sprouts [11], and of phenolic compounds in carrots [12]. Moreover, other less-energetic regions of the electromagnetic spectrum have also been shown to be good elicitors of such bioactive compounds, without the potential quality affectation in fruit and vegetables due to the high energetic UV radiation, which may induce cellular damage.

In this sense, this work elucidates the latest research about using green technologies such as UV and visible spectrum lighting to improve the quality of young plants in their first stages of development, enhancing their phytochemical compounds while reducing costs and energy.

## 2. Elicitor’s Classification: Biotic and Abiotic

Horticultural crops are subjected to a wide range of stresses, which can reduce their yield and quality. Such stressors can be abiotic (UV, light, floods, drought, heavy metals, salinity, extreme temperatures, wounding, etc.) or biotic (bacteria attacks, fungi, nematodes, insects, and little animals, among others). Their uncontrolled application is one of the main causes of worldwide crop losses [13]. Nevertheless, when some of these stressors are applied in a customised way and under controlled conditions, such exogenous agents stimulate and generate signalling pathways in plants, which are translated into desired cellular responses.

Currently, a compound acts as an elicitor when it can stimulate by itself any kind of plant defence. Therefore, plan elicitors can be described as exogenous stressors (abiotic or biotic), which can enhance the production of secondary metabolites under indirect action, such as phenolic acids, flavonoids, and carotenoids, among other pigments, and glucosinolates and/or their derivatives: isothiocyanates. Although pathogenic bacteria were described as the first biotic elicitor [14], nowadays the essential molecule that produces the desired effect is directly added to generate such stress. Specifically, biotic elicitors activate different signal pathways to synthetise secondary metabolites as defensive molecules.

Independently from their origin, stimuli caused by stressors are received from the sensory receptors located on the cellular wall and are transferred throughout the cytoplasm to the nucleus, which triggers transcriptional changes that helps the plant to tolerate the suffered stress. In consequence, the defence against a pathogen attack includes the triggering of physiological barriers, proteins, and enzymes that confer the resistance against such stress.

Nevertheless, the application of biotic elicitors from pathogen microorganisms is not an attractive way of enriching horticultural commodities for human consumption. Therefore, abiotic stressors, which share an inert and mainly technological origin, have been developed in last few decades to be applied during growing and after harvesting. In this sense, to enhance the biosynthesis and accumulation of nutraceuticals, Cisneros-Zevallos [15] in 2003 first proposed the application of controlled postharvest abiotic stresses, which was recently updated in 2020 [16], and divided it into physical and chemical elicitors.

Physical elicitors are external agents applied under control to the plant in the form of physical damage, which can be produced by wounding, gas composition (modified atmospheres), temperature, humidity, salinity, osmolarity, or lighting. For instance, wounding carrot tissues has been shown to be an interesting way to increase the content of phenolic acids, mainly chlorogenic acid, especially combined with temperatures higher than 15 °C, UV-C lighting, and/or the application of methyl jasmonate, which can be a consequence of the activation of transcriptomic genes involved in the biosynthesis of phenolics [13,17]. In addition, the enzyme phenylalanine ammonia lyase (PAL) is activated by abiotic stresses against reactive oxygen species (ROS) accumulation, which is linked to the production of phenolic compounds [13,17]. In fact, the wounding of fresh carrot has been demonstrated to be effective also in combination with UV-A, UV-B, and UV-C lighting, whose application has been related to the production of ROS and, consequently, to the production of antioxidant compounds to fight against such oxidative processes [18,19]. In this sense, a direct correlation has been found between the UV-B application and the biosynthesis of nutraceuticals, which was recently corroborated in red prickly pears [20], broccoli by-products [21], carrots [22], and red bell peppers [8,23] to enhance the accumulation of glucosinolates and sulforaphane, phenolics, and carotenoids, respectively. These studies showed that the external stress provoked by UV increases the transcription of genes (FMO GS-OX5 -flavin-monooxygenase glucosinolate S-oxygenase 5- and MYB51) involved in the biosynthetic pathway of glucosinolates [2], as well as the genetic code in charge of the secondary metabolism to counteract the ROS action when UV RESISTANCE LOCUS 8 (UVR8) is activated [24].

Furthermore, besides its use as a sanitiser, UV-C in low doses has been shown to be an elicitor of the biosynthesis of carotenoids and flavonoids in red bell peppers on its own or combined with UV-B [8], and in tatsoi baby leaves combined with hyperoxia conditions [25]. Additionally, as a physical elicitor, modified atmospheres containing high oxygen partial pressure act by enhancing the biosynthesis of phenolics in carrot [26]. These findings have been associated with the direct response of the plant against the oxidation processes produced by such elicitors. In fact, these physical elicitors act throughout the ionisation of the water molecules of the plant cells, which increase the ROS accumulation and the production of natural antioxidants to avoid their effect [27].

Regarding chemical elicitors, inorganic salts, ethanol, ethane, benzothiadiazole, acetic acid, and heavy metals in the appropriate dose can also increase phytochemical production, as is the case in the use of AgNO_3_ or CdCl_2_ to increase the production of alkaloids in crops [28]. Moreover, several plant hormones, such as jasmonic and salicylic acid, methyl jasmonate, methyl salicylate, ethylene, gibberellin, and cytokinin, are considered abiotic stressors to stimulate the accumulation of health-promoting compounds, because they act as transcription factors and mediators in hormone-signalling pathways [9,29].

## 3. UV and Visible Spectrum Light Sources

Illumination is essential for plant growth and development. Artificial light sources have substituted sunlight for many years to optimise crop yields. In this sense, lighting with specific regions of the UV and visible spectrum are nowadays applied in growth chambers, greenhouses, and vertical farming to obtain standardised and healthier fresh fruit and vegetables.

LEDs are diodes that only allow current to flow in one direction, called the forward direction. A solid diode is made by adding several layers of silicon, but a diode does not emit light by itself. LEDs emit light due to their components In, Ga, and N, which are mixed and, thanks to the current, emit bluish light. On the other hand, by mixing Al with In and Ga, we get a reddish light. When we mix several of these components in the layers of solid diodes and give them electricity, we get them to light up [30]. With different mixtures of the above components, different colours are achieved, but not all of them.

Particularly, LEDs have several advantages that makes them attractive for all their applications. For instance, their use supposes a decrease of 80% of the energetic cost compared with halogen lights, besides their long life (>100,000 h) without any maintenance. As a matter of fact, the lighting yield of LEDs, which is the electric energy converted to visible light, is 75% higher than conventional halogen lights with 15%, as well as the chromatic reproduction index (CRI), which increases to 90% and is the quality of the light source to reproduce the colours.

**Figure 1:**
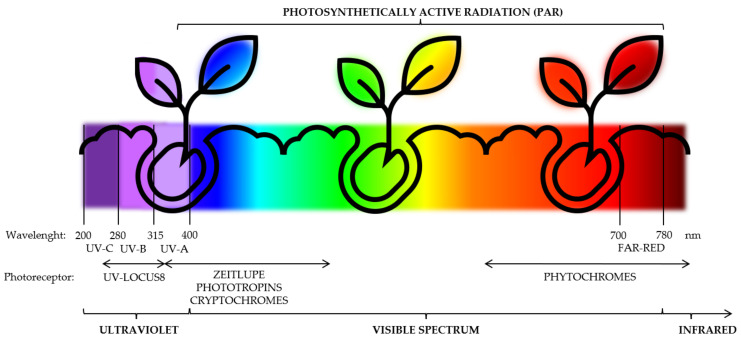
Main photoreceptors implied in light capture according to the UV and visible light spectrum.

In the case of UV light, a UV LED lamp is generally produced on aluminium nitride (AlN) to reach wavelengths lower than 350 nm [31]. New generations of UV LED are more efficient, have a longer service life, save more energy and, hence, protect the environment, despite the high purity of the UV rays emitted by UV LED lamps. Although traditional UV lamps were produced on high-pressure mercury, they produced ultra-high temperatures that are avoided by UV-LED lamps, which do not raise the temperature during the treatment, acting as a cold light source and as a kind of protection for the equipment and for the product [32]. Moreover, the UV LED tube does not use ozone, as high-pressure mercury lamps do, and there are no heavy metal elements inside, which is why it is environmentally friendly [32]. Furthermore, the specific range of emission of the electromagnetic spectrum (UV-C, UV-B, UV-A, and visible spectrum lights) allows it to optimise plant development and the activation of the plant secondary metabolism (Figure 1), acting as an abiotic elicitor of the production of nutraceuticals, especially during the first growth stages, as is the case for sprouts, microgreens, and baby leaves.

## 4. Light Stresses as Elicitors of the Biosynthesis of Phytochemicals: Mode of Action

**Figure 2:**
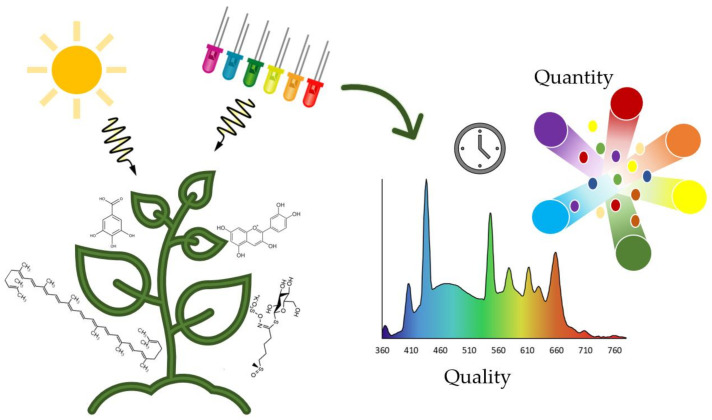
Key light factors influencing the plant secondary metabolism to produce bioactive compounds.

Elicitation of the biosynthesis of bioactive compounds as a defence response against light incidence depends on key factors that affect morphology and secondary metabolism activation. In this sense, both light quality and quantity are essential factors to control (Figure 2). As a result, when we talk about light quality, we refer to the wavelength applied as the specific region of the electromagnetic spectrum. From another point of view, the light direction (direct or indirect incidence), the photoperiod (application time), and the intensity are the main factors to study the specific dose, referring to the light quantity applied to the plant (Figure 2). As a result, the physiological effects of LED lighting on plant growth and development can vary according to these parameters. The main physiological effects can lead to the activation of the primary metabolism: respiration, senescence, photosynthesis, sprouting, growth, and blooming.

In this way, when the quantity of UV light (200–400 nm) exceeds the tolerance of the plant, instead of acting as an abiotic elicitor it causes DNA damage, slows photosynthesis, reduces flowering and pollination, and affects seed development. Particularly, UV-A can cause plant elongation, being less energetic than UV-B or UV-C and belonging to the beginning of the photosynthetic active radiation (PAR) spectral range [33]. 

Before the UV section, blue light (400–500 nm) makes photosynthesis more efficient, which is necessary for this process because it is responsible for vegetative and leaf growth, especially for young plants such as sprouts, microgreens, or baby leaves. In addition, it is essential for opening the stomata and reducing the stretching of the plant by the suppression of spreading growth [34]. Therefore, plants grown in blue light tend to be shorter and have smaller, thicker, and darker green leaves compared to plants grown without blue light. In consequence, blue photons drive the photosynthetic reaction, although their high energy is not fully utilised. However, a minimum intensity of blue light is needed for normal plant growth. 

In this sense, radiation with shorter wavelengths, such as blue and UV lights, stimulates the production of pigments that absorb light and influence leaf colouring, such as chlorophylls and carotenoids. Furthermore, in some leafy green crops such as lettuce, blue also increases the production of health-promoting compounds such as antioxidants (phenolic acids, flavonoids, carotenoids, anthocyanins, and anthocyanidins) and some vitamins (E and C) [35,36,37]. Thus, the application of blue and UV radiation prior to marketing can increase crop quality attributes such as leaf colouring and the bioactive content. Likewise, in the absence of blue and UV, some plants from the tomato family develop intumescence, or small blisters, on the leaves, stems, and petioles [38,39]. This physiological disorder usually decreases as blue and UV radiation increases, which has been also tested to demonstrate antifungal activity [39].

At the end of the PAR region, red light (600–700 nm) is the other critical point for light absorption in leaves. The phytochrome inside the leaves is more sensitive and responsive to red light, and it is important in regulating flowering and fruit production. It also helps to increase stem diameter and stimulates branching [40]. Therefore, red light is the second largest contributor to photosynthesis, but similarly to blue it produces unique results in plant physiology. In fact, it can provide high plant growth, but without the limiting effect of blue, which darkens the chloroplast to protect it from the blue midday sun. Therefore, red is very efficient in producing tall, strong, fast-growing plants and, in fact, produces some of the highest growth rates of height and stem width in plants [41]. The phytochrome pigment mediates plant flowering with a photoperiodic response. In general, plants are very sensitive to low intensities of red light, which can control the moment of the flowering and fruit setting [42].

Far-red light can be found in the higher wavelengths of the visible spectrum (700–800 nm), which can cause plant elongation and trigger flowering in plants. The addition of far-red radiation to red light, in roughly similar amounts, is effective in stimulating flowering of a wide range of long-day plants [42,43]. Therefore, lamps designed to regulate plant flowering always emit red and, in some cases, also far-red light. 

**Figure 3:**
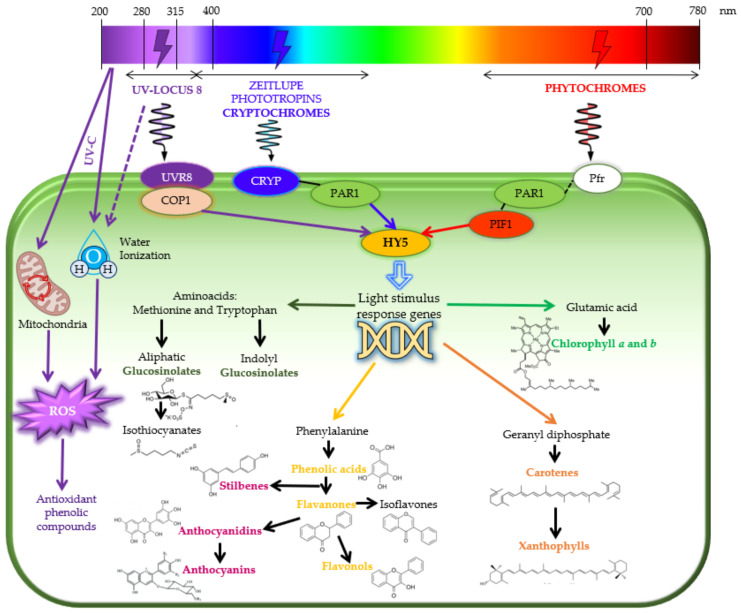
Effect of different wavelengths on plant secondary metabolism: phytochemical accumulation due to photoreceptors and UV-C induced ROS (reactive oxygen species) production. UVR8: UV RESISTANCE LOCUS 8; COP1: COP1 E3 Ubiquitin Ligase; CRYP: cryptochromes; PAR1: t Protease-activated receptor-1; HY5: elongated hypocotyl-5; PIF1: phytochrome-interacting factor-1; Pfr: phototropins.

In addition to physiological and morphological changes, light from different regions of the electromagnetic spectrum is also responsible for mediating genetic responses, which trigger the biosynthesis of chlorophylls, carotenoids, phenolic acids, flavonoids, anthocyanidins, anthocyanins, and glucosinolates (Figure 3).

Specific photoreceptors (Figure 1 and Figure 3), such as UV RESISTANCE LOCUS 8 (UVR8) as the main UV photoreceptor [44]; cryptochromes, phototropins, and zeitlupe family proteins as the UV-A/blue/green photoreceptors [45,46]; and phytochromes as red and far-red photoreceptors [47], are in charge of absorbing light signals that the plant receives. When UV/blue light activates photoreceptors, they activate transcription factors, which link with elongated hypocotyl-5 (HY5) as the main phytoene synthase (PSY) stimulator [48]. Phytochromes also absorb red and far-red wavelengths and interact with the phytochrome-interacting factor (PIF) to trigger the genetic response to stimulate the plant secondary metabolism [48,49]. Furthermore, there is a specific region of the UV-C narrow band (200–250 nm) that does not share UV photoreceptors (UVR8) with the UV-B section. Therefore, the phytochemical accumulation induced by low UV-C doses, which acts as electron donors, has been mainly associated with a mitochondrial energy-dissipating system, and with a partial water molecule ionisation due to it being the most energetic UV radiation, which produces reactive oxygen species (ROS) throughout the mitochondrial electron transport chain for the primary and secondary signalling pathways under UV-C ray incidence [27] (Figure 3).

Accordingly, these reactions are the focus of the main illumination strategies with relevant interest for their application during crop growing and during the postharvest period.

## 5. Preharvest UV and Visible Spectrum Illumination Strategies to Enhance Phytochemicals in Young Plants

Conventionally, the preharvest application of UV and visible spectrum lighting was studied early. A summary of the main effects of UV and visible spectrum illumination in young plants such as sprouts, microgreens, and baby leaves is shown in Table 1. Since the list of scientific publications found about the application of UV and visible spectrum illumination during plant growing is extensive, we just included the 10 most relevant studies in the first stages of plant development (Table 1), based on our opinion. It is remarkable that the main advances in this field were conducted in the last five years. Regarding the use of UV and visible spectrum LED lighting in sprouts, 65% of the references found were published in the last year (2021), which makes this field a hot topic to further elucidate all published hypotheses and reach new findings.

Primarily, Nam et al. [50] compared the biosynthesis of flavonoids and antioxidant compounds under 35 μmol m^−2^ s^−1^ of blue (460 nm), red (625 nm), and fluorescent light in buckwheat sprouts for 7 days. The photoperiod used under blue LED demonstrated the highest accumulation of antioxidant compounds, with significant differences compared to the growth under fluorescent or red lighting. Han et al. [51] carried out a similar study in wheat sprouts, the results of which showed that 82 µmol m^−2^ s^−1^ blue LED was enough to achieve the maximum leaf parameters and enhance bioactive pigment content (carotenoids and chlorophylls). Similar results were also found by Park et al. [52], who studied blue LED in contrast with red and white lighting (50 µmol m^−2^ s^−1^), results of which showed the highest accumulation of phenolics under blue LEDs and the longest development under red LEDs. Hernández-Cánovas et al. [53] studied the use of white LED (400–700 nm) on its own or combined with methyl jasmonate, and showed that 230 µmol m^−2^ s^−1^ white LED illumination was able to increase the biosynthesis of glucosinolates in sprouts, as is the case of glucoraphanin in broccoli, dehydro-erucin in radish, hydroxybenzyl-glucosinolate in mustard, and glucoerucin in red cabbage. Yang et al. [54] reported important accumulations of phenolics, flavonoids, and glucoraphanin in broccoli under 50 µmol m^−2^ s^−1^ of blue LEDs on their own and/or combined with red LED lighting (50:50). These illumination treatments (white (W), blue (B), and red (R)) were also studied in radish, soybean, mung bean, and pumpkin sprouts for 5 days by Mastropasqua et al. [55], whose results showed that under 110 µmol m^−2^ s^−1^ of blue, red, and white, all the sprouts preserved the vitamin C, carotenoid, chlorophyll, and anthocyanin content. 

**Table 1:** Recent most relevant preharvest UV and visible spectrum lighting as phytochemicals elicitors in sprouts, microgreens, and baby leaves.

Light Stimuli	Species	Germination Conditions	Light Conditions	Major Findings	Ref.
**Sprouts**					
FL (400–680 nm), B (450 nm), R (660 nm), and FR (730 nm)	Carrot	7 d in darkness + 10 d under photoperiod (16 h light/8 h darkness)	FL: 2016 kJ m^−2^, B + R (25% + 75%): 2073.74 kJ m^−2^, B + R + FR (22% + 65% + 13%): 2361.8 kJ m^−2^	Phenolic content increased by 45% and 65%, and carotenoid content increased by 279 and 220% after B + R and B + R + FR, respectively.	[56]
UV-B (280–320 nm)	Red cabbage	10 d in darkness + UVB on days 3, 5, 7, and 10	UVB5: 1.25 kJ m^−2^ d^−1^, UVB10: 2.50 kJ m^−2^ d^−1^, UVB15: 3.25 kJ m^−2^ d^−1^	UVB15 induced the highest carotenoid content and total antioxidant capacity. Total flavonoid content was increased by 35 and 30% under UVB10 after 10 d at 4 °C.	[57]
UV-B (280–320 nm)	Kale	10 d in darkness + UVB on days 3, 5, 7, and 10	UVB5: 1.25 kJ m^−2^ d^−1^, UVB10: 2.50 kJ m^−2^ d^−1^, UVB15: 3.25 kJ m^−2^ d^−1^	Hydroxycinnamic acid content was increased by 52% and glucoraphanin by 36% after UVB15, even after 10 d at 4 °C.	[11]
W (380 nm), B (470 nm), R (660 nm), and B + R	Canola	14 d at 25 °C under photoperiod (16 h light/8 h darkness)	PPFD: 50 µmol m^−2^ s^−1^	The highest total phenolics under B light. R light increased the sprout growth.	[52]
UV-A (365), B (450 nm), R (660 nm), and combinations	Broccoli	36 h in darkness + 5 d photoperiod (16 h light/8 h darkness) 23 °C and 77% RH	PPFD: 50 µmol m^−2^ s^−1^ for B, R, R + B (50 + 50%), and R + UVA (50 + 50%)	B and R + B estimulated cotyledon metabolite increased total phenolic content, total flavonoid content, and glucoraphanin content.	[54]
B (465 nm), R (650–670 nm), and W (400–700 nm)	Wheat	10 d under photoperiod (16 h light/8 h darkness) 23 °C and 70% RH	PPFD: up to 264 µmol s^−1^ for R, 201 µmol s^−1^ for W, 263 µmol s^−1^ for B	A PPFD of 82 µmol s^−1^ was enough to achieve the maximun leaf parameters.	[51]
FL, B (460 nm), and R (625 nm)	Buckwheat	2 d in darkness + 7 d under photoperiod (16 h light/8 h darkness) at 25 °C	PPFD: 35 µmol s^−1^	B light increased total phenolics, total flavonoid content, and antioxidant capacity.	[50]
W (400–700 nm), B (440 nm), G (545 nm), Y (580 nm), and R (610 nm)	Soybean, mung bean, radish, and pumpkin	5 d under photoperiod (16 h light/8 h darkness) 24 °C and 78% RH	PPFD: 110 µmol s^−1^	R light in soybean sprouts increased total phenolics and W, R, and B light preserved vitamin C, carotenoid, chlorophyll, and anthocyanin content in all sprouts.	[55]
W and combinations of B (460 nm) and R (640 nm)	Chinese kale	1 d at 28 °C + 9 d under photoperiods (0–16 h light/24–8 h darkness) 25 °C and 78% RH	PPFD: 150 µmol s^−1^ for W and combinations of B + R (10 + 0%, 8 + 2%, 5 + 5%, 2 + 8%, and 0 + 10%)	B + R (10 + 0%) light increased glucosinolates.	[58]
UV-B (300 nm), B (450 nm), and G (510 nm)	Kale, rocket, and kohlrabi	3 d + 8 d under photoperiod (12 h light/12 h darkness) at 20 °C	PPFD: B 99 µmol m^−2^ s^−1^ or G 119 µmol m^−2^ s^−1^, + UV-B 1.15 kJ m^−2^	B enhanced concentrations of flavonoid glycosides, but G did not. B + UV-B increased singlet oxygen scavenging.	[59]
W (400–700 nm)	Broccoli, red radish, red cabbage, and white mustard	3 d + 4 d under photoperiod (18 h light/6 h darkness) at 20 °C	PPFD: 230 µmol m^−2^ s^−1^	Increase of glucosinolates in all the species studied.	[53]
**Microgreens**					
Combination of B (445 nm), R1 (638 nm), R2 (665 nm), and FR (735 nm)	Kohlrabi, mustard, red pak choi, and tatsoi	10 d under photoperiod (16 h light at 21 °C/8 h darkness at 17 °C) and 50–60% RH	PPFD: 110–545 µmol m^−2^ s^−1^ B (7.5%), R1 (41.3%), R2 (50.3%); FR (0.9)	320–440 umol m^−2^ s^−1^ Larger leaf and higher total anthocyanins, total phenols, and DPPH scavenging capacity.	[60]
-	Broccoli, kale, Tronchuda cabbage, and red cabbage	12 d at 25 °C under photoperiod (16 h light/8 h darkness)	-	Higher glucosinolate content under photoperiod.	[61]
Fl (400–680 nm) and combinations of B (465 nm), R (669 nm), and FR (730 nm)	Purslane	3 weeks at 25–20 °C and 80% RH under photoperiod (16 h light/8 h darkness)	PPFD: 150 ± 5 µmol m^−2^ s^−1^ Fl: 24 h and R + B ratio of 0.74, B + R (25% + 75%; ratio 3), B + R + FR (22% + 65% + 13%; B + R + 15%FR)	Nitrate content was reduced by 81 and 91% under B + R and B + R + FR, respectively. The lowest oxalate content with B + R and B + R + FR. The highest total phenolic content under B + R.	[62]
B (447 nm), G (520 nm), Y (595 nm), O (622 nm), R1 (638 nm), R2 (665 nm), and FR (731 nm)	Mustard, red pak choi, and tatsoi	10 d at 21/17 °C and 50–60% RH under photoperiod (16 h light/8 h darkness)	PPFD: 300 µmol m^−2^ s^−1^ B + R1 + R2 + FR supplemented with 5% of G, Y, and O	G, Y, and O supplemental wavelengths increased total carotenoids in mustard but decreased it in red pak choi. Carotenoid content increased in tatsoi under supplemental Y light.	[63]
Combinations of B (447 nm) and R (660 nm)	Mustard and kale	5 d at 21/17 °C and 60% RH under photoperiod (18 h light/6 h darkness)	PPFD: 250 µmol m^−2^ s^−1^ B + R at 0 + 100%, 10 + 90%, 25 + 75%, 50 + 50%, 75 + 25%, and 100 + 0%	25 and 50% of B sufficiently induced high yields and levels of mineral nutrients.	[64]
Combinations of B (400–499 nm) and R (600–699 nm)	*Brassica*	6–12 d under lighting	PPFD: 150 µmol m^−2^ s^−1^ B + R at 20 + 80% and 80 + 20% and B + G + R at 20 + 10 + 70% and 70 + 10 + 20%	B + G + R at 20 + 10 + 70% showed a positive influence on the growth and morphology of microgreens.	[65]
B1 (455 nm), B2 (470 nm), B3 (505 nm), Y (590 nm), and R (627 nm)	Red pak choi and tatsoi	7 d at 21/17 °C and 55% RH under photoperiod (16 h light/8 h darkness)	PPFD: 200 µmol m^−2^ s^−1^ Pulsed light frequencies at 2, 32, 256, and 1024 Hz with cycle of 50%	High total phenolic content in mustard under all wavelength LEDs at 2, 256, and 1024 Hz. High anthocyanins in red pak choi and tatsoi with 2 and 32 Hz. The highest antioxidant activity in mustard with 32, 256, and 1024 Hz and in red pak choi and tatsoi with 2 Hz.	[66]
Combinations of B (447 nm), G (520 nm), Y (595 nm), O (622 nm), R1 (638 nm), R2 (665 nm), and FR (731 nm)	Mizuna, broccoli, and kohlrabi	10 d at 21/17 °C and 50–60% RH under photoperiod (16 h light/8 h darkness)	PPFD: 300 µmol m^−2^ s^−1^ B + R1 + R2 + FR (14 + 35 + 50 + 1%), B + R1 + R2 + FR (14 + 30 + 50 + 1%) + 5% of G, Y, and O	Y light increased the soluble carbohydrate content and O light increased Fe, Mg, and Ca content in all microgreens. All supplemented lights increased β-carotene in mizuna and ascorbic acid in kohlrabi.	[67]
Combinations B (455 nm), Y (590 nm), and R (660 nm)	Mizuma, pak choi, radish, and mustard	2–3 d under photoperiod (16 h light/ 8 h darkness) at 24 °C and 90% HR + 13–14 d under the same photoperiod at 16 °C and 70% RH	PPFD: 179–197 µmol m^−2^ s^−1^ of B (20.5–58.9%) + Y (4.7–39.5%) + R (74.4–0.6%)	High antioxidant activity (DPPH), total phenolic content, and total flavonoid content increased in glucosinolates.	[68,69]
Combinations of B (445 nm), R1 (638 nm), R2 (665 nm), and FR (731 nm)	Mustard, beet, and parsley	13 d at 21/17 °C and 50–60% HR under photoperiod (16 h light/ 8 h darkness)	PPFD: 300 µmol m^−2^ s^−1^ R1 + R2 + FR (43 + 56.2 + 0.8%) + B(0–33%)	33% of B light increased chlorophylls *a* and *b*, carotenoids, α- and β-carotenes, lutein, violaxanthin, and zeathin content from 1.2- to 4.3-fold. Tocopherols were increased by 1.3-fold with 16% of B light.	[70]
**Baby Leaves**					
Combination of B (455 nm), R1 (627 nm), R2 (660 nm), and FR (735 nm)	Green- and red-leaf lettuce	27 d at 22/18 °C and 60–70% RH under photoperiod (18 h light/6 h darkness)	PPFD: 100–500 µmol m^−2^ s^−1^ (2% FR, 10% B, 44% R1, and 44% R2)	Adequate growth and lower nitrate and nitrite contents with a PPFD 300–400 µmol m^−2^ s^−1^.	[71]
B (455 nm), R1 (627 nm), R2 (660 nm), and FR (735 nm)	Green- and red-leaf lettuce	25 d under different photoperiods at 22/18 °C and 60–70% RH	Photoperiod: 12–24 h of light with a PPFD of 300 µmol m^−2^ s^−1^ (2% FR, 10% B, 44% R1, and 44% R2)	Adequate growth and lower nitrate and nitrite contents with a photoperiod of 16–18 h of light.	[71]
W supplemented with UV-A	Chinese kale	14 d at 24 °C and 65–75% RH under photoperiod (10 h light/14 h darkness) + 10 d in photoperiod (12 h) of UV-A (0–15 W m^−2^)	W PPFD: 300 µmol m^−2^ s^−1^ UV-A PPFD: 0–30 µmol m^−2^ s^−1^	10 W m^−2^ UV-A improved the plant biomass and morphology, and increased antioxidant compounds. 15 W m^−2^ UV-A increased glucosinolate content.	[72]
B1 (430 nm), B2 (465 nm), UV-A1 (380 nm), UV-A2 (400 nm)	Chinese kale and pak choi	10 d under photoperiod with natural light (35 °C/20 °C; day/night) and 35–90% HR + 10 d in sampe photoperiod supplemented with 12 h of B and UV-A	Natural light PPFD: 400–1000 µmol m^−2^ s^−1^. B and UV-A intensity: 40 W m^−2^	B supplementation resulted in higher biomass, better morphological traits, and higher antioxidant compounds. UV-A supplementation enhanced glucosinolate content.	[73]
W (380–780 nm)	Lettuce sprouts, microgreens, and baby leaves	50 d at 20 °C under photoperiod with 16/8 h (day/night)	PPFD: 107–150 μmol m^− 2^ s^−1^	Lettuce cv. Romana under LED provided 648.44 and 833.71% of the total daily intake required of vitamin A. There was an inverse relationship between nitrate and carotenoid contents.	[74]
W, B (468 nm), R (629 nm), G (524 nm)	Spinach	26 d under photoperiod with natural light	PPFD: 26.0 μmol m^−2^ s^−1^	R stimulated biomass production and G and B increased total phenolic content and antioxidant capacity.	[75]
Combinations: UV (380–399 nm), B (400–499 nm), G (500–599 nm), R (600–699 nm), and FR (700–780 nm)	Spinach	28 d at 22 °C under photoperiod with 16/8 h (day/night)	PPFD: 150 μmol m^−2^ s^−1^	Plants were better developed when they were treated with high proportions of R, either with FR and low B or with balanced B, G, and FR.	[76]
W (380–780 nm) supplemented with R, B, and Gray	Spinach	26 d under photoperiod with natural light and covered by colour nettings	PPFD: R 118.4 μmol m^−2^ s^−1^, B 118 μmol m^−2^ s^−1^, Gray 63.18 μmol m^−2^ s^−1^	The total phenolics and the antioxidant capacity were higher under R, which was also maintained after 10 d at 4 °C.	[77]
Warm W (400–780 nm; peak 639 nm) supplemented UV-A (385 nm), B (449 nm), G (526 nm), R (664 nm)	Lettuce	17 d at 22 °C under photoperiod with 18/6 h	PPFD: 200 μmol m^−2^ s^−1^ WarmW + 30 μmol m^−2^ s^−1^ UV-A or 50 μmol m^−2^ s^−1^ B, G, or R	UV-A and B increased the accumulation of secondary metabolites at harvest and R preserved them after 7 d at 5 °C.	[78]
UV-C (280 nm)	Spinach	27 d at 22 °C under photoperiod with 14/8 h natural light	UV-C intensity: 1.5 kJ m^−2^ and 3 kJ m^−2^	UV-C supplementation resulted in higher total phenolic content, antioxidant capacity, and lower mesophilic load, also maintained after 6 d at 5 °C.	[79]
UV-C (280 nm)	Spinach, whose seeds were inoculated with *Alternaria alternata*	29 d at 22 °C under photoperiod with 14/8 h natural light	UV-C intensity: 0.3, 0.6, and 0.9 kJ m^−2^ × 2 or 5 times	The lower UV-C treatment showed the highest vitamin C content and FRAP antioxidant capacity. The highest UV-C dose reduced the yeast counts.	[80]

UV-A: ultraviolet-A; UV-B: ultraviolet-B; UV-C: ultraviolet-C; FL: fluorescent lights; B: blue LED; G: green LED; Y: yellow LED; R: red LED; FR: far-red LED; W: white LED; PPFD: Photosynthetic Photon Flux Density.

When compared to fluorescent lights, the development of carrot sprouts under blue + red or blue + red + far-red increased the accumulation of phenolic compounds and carotenoids, even two-fold compared to darkness conditions and fluorescent lighting [56]. In addition, in the last year, purslane microgreens were shown to be richer in flavonoids, phenols, carotenoids, and chlorophylls after the same combination of B + R + FR at 150 µmol m^−2^ s^−1^ PPFD for 21 days [62]. This combination of blue + red was also studied by Chen et al. [58] in Chinese kale sprouts, who showed that 150 µmol m^−2^ s^−1^ R LEDs increased the development of kale sprouts and 150 µmol m^−2^ s^−1^ B LEDs enhanced the accumulation of glucosinolates in comparison with the same dose of white or blue + red combinations. In Fiutak et al.’s study [81], apart from obtaining the highest yield with cold and warm LEDs (even 10-fold low cost), regarding fluorescent lights and traditional bulbs, LEDs showed the highest amounts of chlorophylls, β-carotene, lutein, neoxanthin, thiocyanates, and ascorbic acid content in kale sprouts [81].

Regarding UV applications, Lim et al. [82] studied the effect of 24 or 120 h of 2 W m^−2^ UV-A (370 nm) on the accumulation of isoflavones and flavonols in soybean sprouts, which positively affected kaempferol glycosides accumulation. In the same matrix, the authors applied 24 or 36 h of 2 W m^−2^ UV-B (314 nm), which positively affected the isoflavone accumulation and the gen stimuli related to chalcone synthases, mainly related to the flavonoid biosynthesis [83]. When UV-B and coloured LEDs were combined, Neugart et al. [59] showed that 99 µmol m^−2^ s^−1^ blue LEDs increased and stabilised the flavonoid content in kale, kohlrabi, and rocket sprouts, whereas 119 µmol m^−2^ s^−1^ green LEDs supplemented with UV-B 1.15 W m^−2^ did not. Gui et al. [84] studied the use of UV-B daily treatments (0.7 W m^−2^) during the growth of mung bean sprouts and reported an inhibition of the microbial growth. 

Martínez-Zamora et al. [57] developed a UV-B dose-dependence model in red cabbage sprouts, in which the highest concentrations of flavonoids, phenols, and carotenoids were obtained after periodical UV treatments during growing, with a total dose of 5, 10, and 15 W m^−2^. Similarly, Castillejo et al. [11], following the same treatments, obtained the highest concentration of phenolic compounds and glucosinolates with a total dose of 10 and 15 W m^−2^ in kale sprouts. Such results demonstrated that the periodical application of low UV-B doses during growing can optimise the bioactive content of young plants.

Regarding microgreen development under UV and/or visible spectrum LED lighting conditions, Brassicaceae is the most studied plant family due to its high glucosinolate and isothiocyanate contents. As a matter of fact, 80% of the scientific studies found were performed using brassica microgreens as a model.

Firstly, the application of conventional light during the last days of the germination was shown to increase the biosynthesis of glucosinolates in brassica microgreens [61]. Before that, Kopsell et al. [85] studied the combination of blue (470 nm) and red (627 nm) LED lighting (350 μmol m^−2^ s^−1^) in a photoperiod for 13 days, which was followed by 5 days of blue:red 350 μmol m^−2^ s^−1^ or B 41 μmol m^−2^ s^−1^ in broccoli. The obtained results shown as the single application of blue LEDs resulted in significant increases of the carotenoids (specially β-carotene by ~40%), glucosinolates (specially glucoraphanin by ~30%, the main source of sulforaphane), and essential minerals from the primary metabolism (K, Mg, and Fe by ~100%). After that, the same authors supported their own results with a study of the combination of narrow-band spectra (blue (447 nm), red (627 nm), and green (530 nm)) at different proportions [86]. Results showed that under higher proportions of blue (20%) and red (80%) LED lighting, broccoli microgreens accumulated a higher proportion of chlorophylls (+150%), carotenoids (+35%), glucoraphanin (+200%), and minerals (+100%) compared to fluorescent lighting, which corroborated previous findings by the authors. Brazaityte et al. [64] corroborated that a high percentage of blue light, specifically 75 or 100% of the total light applied (250 μmol m^−2^ s^−1^ in an 18 h photoperiod for 5 days), positively affected the accumulation of nutrients in young Brassicaceae plants, which could be strategically used as a tool for mustard and kale microgreen biofortification through the increase of chlorophyll, flavonol, anthocyanin, and carotenoid accumulation.

Samuoline et al. [60] studied how the combination of blue, red, and far-red at different intensities (from 100 (545 μmol m^−2^ s^−1^) to 20% (110 μmol m^−2^ s^−1^)) affects the growth and nutritional quality of brassica microgreens (kohlrabi, mustard, red pak choi, and tatsoi) for 10 days. The obtained results showed that the best conditions for growth and antioxidant activity were reached under 330–440 μmol m^−2^ s^−1^, which resulted in larger leaf surface area, lower content of nitrates, and higher content of anthocyanins, phenols, and antioxidant capacity. By contrast, a lower dose did not reach the minimum nutritional quality of the brassica microgreens and a higher dose did not show significant differences with the cited intensities. Posteriorly, the same authors demonstrated that the incorporation of blue light at different doses (25–33%) into the light spectrum (combined with red and far-red, with a total PPFD of 300 μmol m^−2^ s^−1^), applied during the growth of mustard, beet, and parsley microgreens, can enhance the biosynthesis of carotenoids and tocopherols of those species [70]. In addition, in 2019, these authors studied a similar light combination in broccoli, mizuna, and kohlrabi, which was supplemented with yellow, green, and orange lights to preserve the mineral content (Fe, Mg, and Ca) and sugars, and increase the biosynthesis of carotenoids and ascorbic acid [67], especially with the incorporation of the yellow region to the light spectrum. Also in brassica microgreens, Alrifai et al. [69] recently showed that the incorporation of yellow light (590 nm) during preharvest of mizunas, pak choi, red radish, and white mustard is able to improve the glucosinolate profile in combination with standard blue and red lights to modulate the pathway biosynthesis of the precursors of the isothiocyanates. In a similar experiment, these authors also demonstrated this beneficial effect on the biosynthesis of phenolic compounds under the same illumination on the same brassica cvs [68], which was positively related to their antioxidant capacity.

Although individual supplementation with blue and red lights turned out to be more efficient than yellow light in mustard, red pak choi, and tatsoi to enhance the antioxidant capacity and phenolic compound biosynthesis [66], recent findings by these authors demonstrated a similar light combination in broccoli, mizuna, and kohlrabi, which was supplemented with yellow, green, and orange lights to preserve the mineral content (Fe, Mg, and Ca) and sugars, and increase the biosynthesis of carotenoids and ascorbic acid [67], especially with the incorporation of the yellow region to the light spectrum. Furthermore, these authors specified that the best dose to optimise the biosynthesis of carotenoids was 330–440 μmol m^−2^ s^−1^ in red pak choi and tatsoi and in mustard 110–220 μmol m^−2^ s^−1^ during growth for 10 days [63].

Moreover, the supplementation with 10% green light with 70% red and 20% blue showed a positive influence on the growth and morphology of brassica *cvs* microgreens of kohlrabi, red cabbage, broccoli, kale, komatsuna, tatsoi, and green cabbage, which translated into higher carotenoids and vitamin C content [65].

Regarding baby leaf development under different light conditions, all the studies found were published in last three years, with 50% in 2021, 20% in 2020, and 30% in 2019. The most studied varieties were spinach baby leaves, followed by lettuce and Brassicaceae baby leaves. Viršilè et al. [71] associated the nitrate assimilation by red and green baby-leaf lettuces with different intensities (100–500 μmol m^−2^ s^−1^ of blue, red, and far-red) and photoperiods (12–24 h), establishing that for an efficient nitrate assimilation a range of 300–400 μmol m^−2^ s^−1^ and a minimum photoperiod of 16–18 h are needed. This nitrate assimilation is directly related to the optimum growth parameters and chlorophyll content of both varieties. Vaštakaitè-Kairienè et al. [78] studied the characteristics of baby-leaf lettuce growth under 200 μmol m^−2^ s^−1^ of warm white lights and supplemented with 30 μmol m^−2^ s^−1^ UV-A or 50 μmol m^−2^ s^−1^ blue, 50 μmol m^−2^ s^−1^ green, 50 μmol m^−2^ s^−1^ red, or 50 μmol m^−2^ s^−1^ warm white LEDs. The obtained results showed that the different supplementations did not affect the physiological development, whereas green LEDs increased the total antioxidant activity (measured by DPPH), blue LEDs increased the accumulation of secondary metabolites at harvest, and red LEDs preserved the phytochemicals during a short postharvest storage. Ferrón-Carrillo et al. [74] studied the use of different LED lighting, which had a high percent of blue light and a gradual percent of red light. After 50 days and using as sampling times the different stages of lettuce development (sprout, microgreen, and baby leaf), the obtained results showed that treatments with a higher percent of red light increased their vegetative growth, whereas the treatments with a higher percent of blue and red light increased their concentration in carotenoids and preserved it throughout the study.

Regarding Brassicaceae baby leaves, Li et al. [73] and He et al. [72] studied how supplementation with different UV-A doses can affect the growth and nutritional content of such species. The results obtained by these authors showed that both UV-A [72] and blue LED [73] supplementation can increase the glucosinolate content in Chinese kale baby leaves [72], as well as the chlorophylls, carotenoids, anthocyanins, phenolics, vitamin C, and flavonoid (kaempferol and quercetin) content in Chinese kale and pak choi baby leaves [73]. Therefore, under blue LEDs, both brassica baby leaves increased their antioxidant capacity [73].

As previously said, the influence of UV and visible spectrum LEDs on the development and quality of spinach baby leaves has been widely studied. Battistoni et al. [75] used white, blue, red, and green LEDs for 26 days to increase the nutritional quality of spinach baby leaves. These authors showed that antioxidant capacity and phenolic accumulation were improved under blue LEDs. Moreover, Bantis et al. [76] used different LED lamps that combined UV, blue, green, red, and far-red at different percentages. Under 150 μmol m^−2^ s^−1^ of the treatment with a high percent of red (67%) and far-red (23%) the yield production was significantly increased. Nevertheless, the LEDs with 0.02% UV, 11% blue, 14% green, 56.5% red, and 18.5% far-red improved the nutritional quality of the spinach leaves by inducing the production of phenols, chlorophylls, and carotenoids.

In a different way, Lara et al. [77] studied the light incidence by using different shade-netting colours (red: 118.35 μmol m^−2^ s^−1^, blue: 117.96 μmol m^−2^ s^−1^, grey: 63.18 μmol m^−2^ s^−1^, and control without shade nettings: 278.12 μmol m^−2^ s^−1^). As other authors corroborated by using LED lighting, the use of the blue netting enhanced the biosynthesis of phenolics, whereas the use of the red netting increased the antioxidant activity of spinach baby leaves.

Furthermore, the use of UV-C at low doses (0.3, 0.6 and 0.9 kJ m^−2^) has been demonstrated to reduce the antifungal activity in spinach baby leaves by 3 log CFU g^−1^ [79,80]. Particularly, the vitamin C and phenolics contents were improved, which was related to an increased antioxidant capacity. In this sense, these nutritional quality improvements of spinach baby leaves can be justified by the contrast of antioxidants produced as electron donors versus ROS production under UV-C illumination (Figure 3), as previously described Rabelo et al. [27]. 

During growth, specific photoreceptors (Figure 1 and Figure 3) absorb light signals that the plant receives and activate the genetic pathway to stimuli HY5 and PSY, mainly responsible for the secondary metabolism and initiators of the biosynthesis of carotenoids, flavonoids, and phenolics [48].

As shown, the different regions of the visible spectrum used in a customised and controlled way have shown important beneficial effects in the development and nutritional quality of sprouts, microgreens, and baby leaves. Their low cost and long durability make the incorporation of UV and visible spectrum LED gadgets economic and technological viable, especially in vertical farming and greenhouses, where the preharvest conditions can be monitored, resulting in increased crop yields.

## 6. Postharvest UV and Visible Spectrum Illumination Strategies to Enhance Phytochemicals in Young Plants

In the last 50 years, postharvest technologies have gained special attention for prolonging the shelf life of horticultural commodities. Nevertheless, the use of UV and visible spectrum lighting strategies in young plants during their refrigerated shelf-life period, mainly as minimally processed products, is still quite limited. It is remarkable to see that only 13 manuscripts have been published in this field, and all of them in the last five years. Accordingly, Table 2 summarises the conditions used and main findings of such works regarding young plants such as sprouts, microgreens, and baby leaves.

Moreira-Rodríguez et al. [2,9] showed that UV-A and UV-B increased the accumulation and biosynthesis of secondary metabolites such as phenolics, carotenoids, flavonoids, and glucosinolates in broccoli sprouts after 24 h. In the first study [2], when they studied the application of low and high doses of UV-A (3.16 and 4.05 W m^−2^) and UV-B (2.28 and 3.34 W m^−2^), they observed positive stimuli on the biosynthesis of phenolics and glucosinolates. As a matter of fact, a dose-dependence behaviour was shown in the derivatives of gallic and gallotanninc acid, 4-*O*-caffeoylquinic acid (derivative of chlorogenic acid), and 5-sinapoylquinic acid (derivative of sinapic acid). Moreover, high doses of UV-B showed relevant increases of aliphatic (glucoiberin, glucoerucin, and glucoraphanin) and indolyl glucosinolates (glucobrassicin and 4-methoxy-glucobrassicin), the first being those from which isothyocyanates are derived. Although in this study a shelf-life study was not performed, 24 h after the UV-B treatment, increases of ~170–75% in the concentration of aliphatic glucosinolates and ~120–50% in phenolic compounds were shown. This increase in the glucosinolate accumulation was related to the stimuli of the genetic expression from aliphatic (FMO GS-OX5) and inolyl glucosinolates (MYB51) [2,9], whose biosynthesis is also enhanced by the activation of the photoreceptor UVR8, when it interacts with COP1 to induce the transcription factor HY5 (Figure 3).

Using higher doses (9.47 W m^−2^ UV-A or 7.16 W m^−2^ UV-B) and studying the combination with methyl jasmonate, in their second study [9], the authors showed that the application of UV-B alone can increase the biosynthesis of phenolic compounds, chrolophylls, carotenoids, and glucosinolates in broccoli sprouts 24 h after the treatment. Hence, glucosinolate content was increased by ~78–177%, whereas carotenoids and chlorophylls were biostimulated by ~100% and phenolic content by ~25–75%.

More recently, our research group corroborated this behaviour by applying higher doses of UV-B and its subsequent preservation effect during a refrigerated shelf-life period in broccoli and radish sprouts [10]. In this case, 15 W m^−2^ UV-B and/or 9 W m^−2^ UV-C were applied after harvesting in individual and simultaneously combined treatments, and UV-B showed to be an important elicitor of the total antioxidant capacity, the total phenolic content, and the glucosinolate compounds, which increased by ~35%. In addition, this effect was translated to a ~75% increase of sulforaphane and sulforaphene, whose concentration was 60-fold higher in radish than in broccoli sprouts. Our research group also demonstrated the positive effect of moderate doses of UV-B and UV-C in red bell peppers and the biosynthesis of flavonoids and carotenes [8], which is also effective in such fruits in combination with blue and red LED lighting [23]. Such an elicitation is also induced by longer wavelengths of the visible spectrum. For instance, postharvest storage of broccoli sprouts under continuous 35 μmol m^−2^ s^−1^ red and far-red LED lighting has shown to be more effective for the biosynthesis of phenolic compounds than blue LEDs [87]. Furthermore, although the major effects of LED lighting on the biosyntheis of bioactive compounds were related to the blue region, a continuous 35 μmol m^−2^ s^−1^ yellow illumination was effective for enhancing and maintaining the biosynthesis of glucosinolates (+85%) and phenolic compounds (+ 35%) for 15 d under refrigerated storage, even more so than green and white LED lighting conditions [88]. This behaviour may be due to the activation of key enzymes (MAM1, CYP79F1, CYP83A1, CYP83B1, SUR1, UGT74B1, MAM1, CYP79F1, and CYP83A1) related to the glucosinolate biosynthesis and that can be activated by the yellow region of the visible spectrum [88].

Regarding light incidence during the shelf life of microgreens, Xiao et al. [90] studied the quality of radish microgreens under 30 μmol m^−2^ s^−1^ continuous fluorescent light and reported a high maintenance of vitamin C and total antioxidant capacity. Nevertheless, the direct and continuous exposure to fluorescent light increased weight loss during storage and accelerated the deterioration of radish microgreens. Better findings were obtained in broccoli sprouts combining CaCl_2_ spray and 0.18 and 0.36 W m^−2^ UV-B fluorescent light treatments [89]. Lu et al. [89] focused their work on the increase of the main bioactive compounds (glucosinolates) through exposure to UV-B on its own or combined with a preharvest application of CaCl_2_ spray. The results obtained by these authors showed that UV-B and/or 10 mM CaCl_2_ spray was able to increase the proportion of the main aliphatic glucosinolates by 90%, indicating that it is an effective method to extend the shelf life of broccoli microgreens.

In this sense, postharvest illumination of baby leaves has also resulted in good perspectives about this ‘green technology. For instance, Sun et al. [96] showed that only the continuous exposure of mustard baby leaves to 36 μmol m^−2^ s^−1^ fluorescent light for 6 d at 20 °C was able to increase the biosynthesis of health-promoting compounds, such as ascorbic acid, flavonoids, total phenolic compounds, and glucosinolates, while significantly delaying quality deterioration.

Regarding visible spectrum LEDs, Pennisi et al. [91] showed a positive correlation between LED lighting and the accumulation of nutraceuticals in minimally processed red chard and baby rocket leaves. In this study, continuous 35 μmol m^−2^ s^−1^ white, blue, green, yellow, red, and far-red illumination was individually applied for 10 d at 5 °C. The results obtained showed an increase in the total polyphenol, chlorophyll, and carotenoid content under blue, green, yellow, and red LEDs compared to white, far-red, and darkness conditions, which was translated into a higher antioxidant capacity for both species. These authors also obtained higher weight losses under lighting, as Xiao et al. [90] reported, but these losses were not related to the loss of the bromatological quality.

Furthermore, 5–20 μmol m^−2^ s^−1^ white LED lighting application in pak choi baby leaves for 7 d at 20 °C was demonstrated to maintain the levels of gene expression and antioxidant enzyme activity while increasing the expression of the chlorophyll synthetase gene [92]. Similarly, Yan et al. [94] showed that low treatments in baby pak choi leaves with white LEDs (91 μmol m^−2^ s^−1^) for 5 d at 20 °C stimulated the biosynthesis of glucosinolates and chlorophylls, as well as the gene expression that regulates the photosynthesis and the accumulation of such compounds. Song et al. [93] studied the application of monochromatic lights of the visible spectrum, such as blue, red, and far-red (35 μmol m^−2^ s^−1^), for 5 d at 20 °C. Red LEDs allowed the promotion of gene expression, which triggered the biosynthesis of vitamin C and chlorophylls, which resulted in a delay of the senescence of baby pak choi leaves. By contrast, far-red LED lighting enhanced the leaf senescence. Although in the latest study [93] blue LED lighting did not lead to interesting findings, Jin et al. [95] showed that, under refrigerated conditions, blue LEDs (30 μmol m^−2^ s^−1^) can increase the shelf life of amaranth baby leaves for an additional 2–3 d at 4 °C, increasing the antioxidant capacity when compared to lower doses, such as 10 and 20 μmol m^−2^ s^−1^.

As studied, UV and visible spectrum LED lighting have been demonstrated to increase the functional quality of young plants in their first stages of development, such as sprouts, microgreens, and baby leaves, which are well known for their natural high content in health-promoting compounds. This review summarised the most relevant and novel findings after preharvest and postharvest applications of UV and visible spectrum LED illumination treatments. Furthermore, such illumination technologies have been shown to be useful to increase the phytochemical content in adult plants [97], in vegetable-based beverages [98], and in by-products obtained from the agri-food industry [99], which demonstrates the great potential of such strategies.

## 7. Conclusions and Future Directions

Preharvest UV and visible spectrum LED illumination has been widely developed in recent years to increase the crop yield, especially in vertical farming, where the conditions can be customised and monitored throughout the growing period to obtain phytochemical-enriched products with reproducible quality attributes. Although the literature regarding the postharvest application of such treatments in sprouts, microgreens, and baby leaves is scarce, an extensive field is still open to research the advantages of postharvest UV and visible spectrum LED lighting application, especially in the first stages of plant development. As a matter of fact, specific wavelengths of the electromagnetic spectrum (from 200 to 800 nm), applied in specific conditions, are able to trigger the reactions in the genetic chain to increase the biosynthesis of health-promoting compounds to fight against the external stress produced. 

We are currently far from understanding the mechanistic responses and their dependency on the illumination conditions (wavelengths, combinations, time, dose, development stage, photoperiod, etc.). Whether the signalling involved in the responses of young plants to specific illumination conditions is systemic, local, or both is still debated, without a clear response. Although the scientific community has recently made great progress in understanding the mechanisms, further works should be conducted to elucidate what we still should know, including issues from the physiological, biochemical, and technological points of view.

Furthermore, their low cost and energy saving make their use easy and technologically viable. Such illumination technologies seems to be a promising tool in the horticultural industry, especially during growing in vertical farming, but also after harvesting, during the commercial life (transportation in refrigerated trailers/containers, retail life in supermarkets, and even on consumers’ fridges at home), which has been unexplored. However, problems regarding achieving a uniform light intensity to reach the whole packed commodity during shelf life are still unsolved. 

## Figures and Tables

**Table 2:** Postharvest UV and visible spectrum lighting as elicitors of phytochemicals and preservative techniques for sprouts, microgreens, and baby leaves.

Light Stimuli	Light Conditions	Species	Shelf Life	Major Findings	Ref.
**Sprouts**					
B (465 nm), R (660 nm), FR (730 nm)	35 μmol m^−2^ s^−1^	Broccoli	15 d 5 °C 90% RH	R and FR increased the sprout length and decreased the microbial load.	[87]
W (610 nm), G (517 nm), Y (600 nm)	35 μmol m^−2^ s^−1^	Broccoli	15 d 5 °C 90% RH	W and Y increased phenolic accumulation (+86%). Y increased glucosinolate accumulation (+84%). G slightly increased the accumulation of glucosinolates.	[88]
UV-B (394 nm), UV-C (245 nm)	2 doses of 7.5 W m^−2^ UV-B and/or 4.5 W m^−2^ UV-C	Broccoli Radish	10 d 4 °C 90% RH	UV-B increased glucosinolate (+38%) and isothyocyanate (+72%) contents in broccoli and radish sprouts, and maintained it during their shelf life.	[10]
UV-A (360 nm), UV-B (300 nm)	9.47 W m^−2^ UV-A or 7.16 W m^−2^ UV-B	Broccoli	1 d at room temperature	UV-A and UV-B increased the accumulation of phenolics, flavonoids, carotenoids, chlorophylls, and glucosinolates.	[9]
UV-A (360 nm), UV-B (300 nm)	3.16 and 4.05 W m^−2^ UV-A or 2.28 and 3.34 W m^−2^ UV-B	Broccoli	1 d at room temperature	High doses of UV-A and UV-B increased the accumulation of phenolics, flavonoids, and glucosinolates.	[2]
**Microgreens**					
UV-B (300 nm)	0.18 and 0.36 W m^−2^	Broccoli	21 d 4 °C 90% RH	UV-B + CaCl_2_ increased glucoerucin and glucosinolates.	[89]
Fl (400–780 nm)	30 μmol m^−2^ s^−1^	Daikon radish	16 d 5 °C	Light exposure increased and mantained the ascorbic acid content.	[90]
**Baby Leaves**					
W (610 nm), B (465 nm), G (517 nm), Y (600 nm), R (660 nm), and FR (730 nm)	35 μmol m^−2^ s^−1^	Red chard and rocket	10 d 5 °C 95% RH	B and Y reduced the microbial load, whereas G and W kept the colour and enhanced the pigment concentration. R and B increased the antioxidant activity.	[91]
W (400–780 nm)	5–20 μmol m^−2^ s^−1^	Pak choi	7 d 20 °C 90% RH	10 μmol m^−2^ s^−1^ delayed senescence by reducing respiration and accumulation of MDA and stimulating antioxidant gene expression.	[92]
B (400–499 nm), R (600–699 nm), and FR (700–780 nm)	10–70 μmol m^−2^ s^−1^ 0–24 h light/24–0 h darkness	Pak choi	5 d 20 °C	35 μmol m^−2^ s^−1^ for 8 h R per day inhibited senescence.	[93]
W (400–700 nm)	91 μmol m^−2^ s^−1^	Pak choi	5 d 20 °C	Chlorophyll and glucosinolate biosynthesis were induced by W.	[94]
B (460 nm)	10, 20, 30 μmol m^−2^ s^−1^	Amaranth	12 d 4 °C 90% RH	Sensory scores and antioxidant capacity were increased, whereas spoilage bacteria *Pseudomonas* was reduced.	[95]
Fl (400–780 nm)	36 μmol m^−2^ s^−1^ 12 or 24 h/12 or 0 h darkness	Mustard	6 d 20 °C 75% RH	24 h light maintained sensory quality, avoided deterioration, and retarded losses of sugars, vitamins, antioxidants, and glucosinolates.	[96]

UV-A: ultraviolet-A; UV-B: ultraviolet-B; UV-C: ultraviolet-C; Fl: fluorescent lights; B: blue LED; G: green LED; Y: yellow LED; R: red LED; FR: far-red LED; W: white LED. MDA: Malondialdehyde.

## Data Availability

Data sharing is not applicable to this article as no new data were created or analysed in this article.
